# Sustained Activation of TNFα-Induced DNA Damage Response in Newly Differentiated Adipocytes

**DOI:** 10.3390/ijms221910548

**Published:** 2021-09-29

**Authors:** Mahara Valverde, Aarón Sánchez-Brito

**Affiliations:** Instituto de Investigaciones Biomédicas, Universidad Nacional Autónoma de México, Mexico City 04510, Mexico; agsb92@gmail.com

**Keywords:** DNA-damage response, TNFα, human adipocytes, γH2AX, pATM, comet assay, hASCs

## Abstract

The response to DNA damage is the mechanism that allows the interaction between stress signals, inflammatory secretions, DNA repair, and maintenance of cell and tissue homeostasis. Adipocyte dysfunction is the cellular trigger for various disease states such as insulin resistance, diabetes, and obesity, among many others. Previously, our group demonstrated that adipogenesis per se, from mesenchymal/stromal stem cells derived from human adipose tissue (hASCs), involves an accumulation of DNA damage and a gradual loss of the repair capacity of oxidative DNA damage. Therefore, our objective was to identify whether healthy adipocytes differentiated for the first time from hASCs, when receiving inflammatory signals induced with TNFα, were able to persistently activate the DNA Damage Response and thus trigger adipocyte dysfunction. We found that TNFα at similar levels circulating in obese humans induce a sustained response to DNA damage response as part of the Senescence-Associated Secretory Phenotype. This mechanism shows the impact of inflammatory environment early affect adipocyte function, independently of aging.

## 1. Introduction

The maintenance of cellular and tissular homeostasis relies on the optimal functioning of the DNA. Hence the importance of precise control of DNA damage repair mechanisms by recruiting repair factors to damaged sites and activating checkpoint regulators to halt cell cycle progression, recognized as DNA damage response (DDR) [1]. DDR is initiated through a series of post-transcriptional modifications responsible for the propagation of the response. Initially, molecular damage “sensors” are required to locate distortions or lesions in the DNA. This census function is realized by kinases such as: ATM (Ataxia Telangiectasia Mutated), ATR (ATM and Rad related), and DNA-PKcs (DNA-dependent Protein Kinase catalytic subunit). These kinases phosphorylate, in a targeted manner, multiple substrates near composed of pro-inflammatory cytokines the site of injury. One of the main substrates is histone H2AX (already phosphorylated it is called γH2AX) [2]. On the other hand, a cell cycle arrest will be induced by the activation of the effector kinases Chk2 and Chk1. In this way, the ATM/Chk2 route will respond predominantly to DSBs (double-strand breaks), while ATR/Chk1 will respond to SSBs (single-strand breaks) or adducts. Recently, the study of the phosphorylation and dephosphorylation kinetics of histone H2AX was proposed to classify DDR into transient or short-term response and a sustained or long-term response [3]. The transient response is associated with a rapid and effective repair of the damage; and prompt dephosphorylation of γH2AX to H2AX. This response usually lasts from minutes to a few hours, while the sustained response involves the persistence of γH2AX and other proteins involved in DDR. This second type is associated with senescence and usually lasts from several hours to days or even months [4]. Even though DDR is activated to preserve genomic integrity, its sustained activation has important repercussions for cell function since it favors senescence processes and cellular dysfunction [5].

DDR uses intercellular communication mechanisms developed to activate an extracellular alarm signal and modulate various processes in neighboring cells [2]. In this way, a stress response is generated throughout the tissue that will allow neighboring cells to be prepared for the stressor [6]. This DDR function occurs through soluble signaling molecules that are collectively referred to as the secretory senescence-associated phenotype (SASP) [7]. SASP will be composed of pro-inflammatory cytokines (i.e., IL-6, IL-8, GRO α, GROβ, MCP-1, TNFα), growth factors (i.e., GM-CS, G-CSF, HGF/S, IGF), metalloproteinases (i.e., MMP-1, -2, and -3), and insoluble extracellular matrix proteins (i.e., collagen, fibronectin, and laminin); although its exact composition will vary considerably depending on the type of cell that secretes it [1,8,9].

Although it is well known that a deterioration in DDR activation, during or after embryonic development, implies the progression of syndromes with considerable clinical characteristics that involve mutations in ATM and NBS1 that lead to neurodegeneration, immune dysfunction, radiosensitivity, and predisposition to cancer [1]. Recent work has shown that sustained activation of this pathway is correlated with the development of metabolic alterations [1,9].

It is in this context and considering that our previous work [10] showed an accumulation of DNA damage and a partial loss of the ability to repair oxidative lesions in DNA through adipogenesis, that the purpose of this work was to determine if adipocytes differentiated for the first time from hASCs have DDR activation. Likewise, to determine whether, after adipogenesis under optimal conditions, in vitro obesogenic stimulation of TNFα [11] can induce sustained activation of DDR to understand how metabolic alterations are gestated in healthy adipocytes and their relationship with SASP, regardless of aging.

## 2. Results

### 2.1. Differentiated Adipocytes from Hascs for the First Time

**Figure 1:**
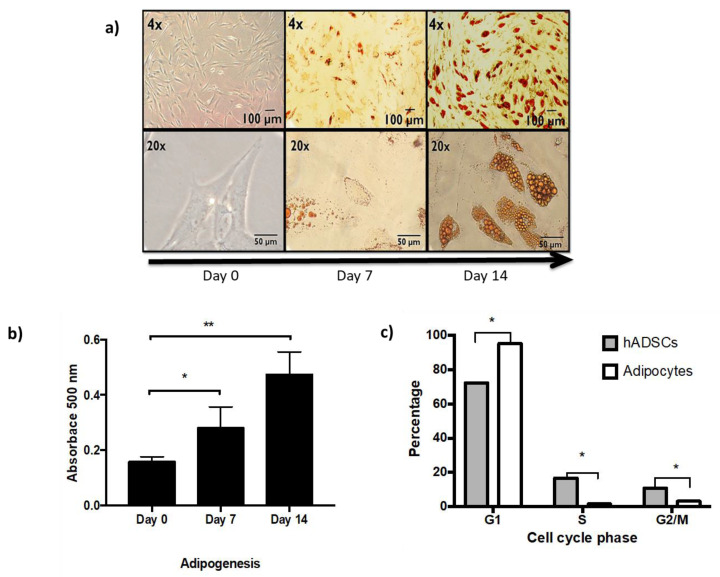
In vitro adipogenesis from hASCs. (**a**) Representative light microscopy images showing Oil Red O staining to identify lipid vesicles acquired through differentiation (day 0, 7, and 14). 4× and 20× magnification of the same fields. (**b**) Spectrophotometric quantification at 500 nm of the Oil Red O staining performed through adipogenesis in three independent experiments, Student’s t test vs control * *p* < 0.05, ** *p* < 0.01. (**c**) Post-mitotic arrest of adipocytes. Percentage of cells in the different stages of the cell cycle, comparing hASCs and 14-day adipocytes. Three independent experiments with experimental replicate were carried out. One way ANOVA test, * *p* < 0.05.

In vitro adipogenesis from hASCs, under optimal conditions, is shown by morphological transition, lipid vesicle secretion, and cell cycle arrest followed for 14 days [Figure 1].

It is possible to identify the change in fusiform morphology and the increase in lipid vesicles from day 7 of differentiation, becoming more evident on day 14 [Figure 1a,b]. While the post-mitotic arrest in the cell cycle of adipocytes with respect to hASCs is shown in Figure 1c. Newly differentiated adipocytes, in a healthy state, are the cell type used for the present study, in which we will show the effect of exposure to TNFα as an obesogenic stimulus in vitro [11].

### 2.2. TNFα Non Affects Cell Viability and Induces DNA Damage

**Figure 2:**
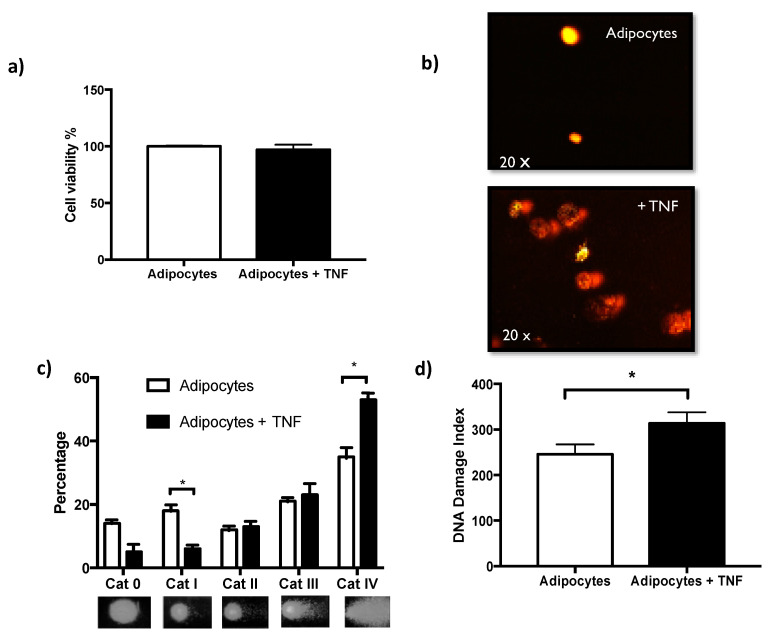
TNFα non affects cell viability and induces DNA damage. (**a**) Cell viability of adipocytes and adipocytes treated by 24 h with TNFα 125 ng/mL, three independent experiments were carried out. Student t test *p* > 0.05 non-significant. (**b**) DNA damage images representatives of comet assay, acquired with *Komet V5.0*. (**c**) Percentage distribution of DNA damage categories (Cat 0 less damage to Cat IV higher damage), in adipocytes and adipocytes treated with TNFα; three independent experiments were carried out. Two-way ANOVA test * *p* < 0.05. (**d**) DNA damage-index differences induced by TNFα treatment, Student’s t test * *p* < 0.05.

Exposure to TNFα for 24 h did not affect cell viability determined by the metabolic activity of esterases [Figure 2a]. This makes it possible to evaluate genotoxicity, ruling out cytotoxic effects. DNA damage determined by the alkaline comet assay is shown by analyzing damage categories. This analysis allows us to identify that TNFα as a stimulus in vitro, has a significant impact on the increased number of comets of category IV, which is the one with the greatest damage. While the control adipocytes show more comets in categories I and II that are the least damaged [Figure 2c]. Alternatively, the results can be seen as a significant increase in the rate of DNA damage induced by TNFα [Figure 2b,d].

**Figure 3:**
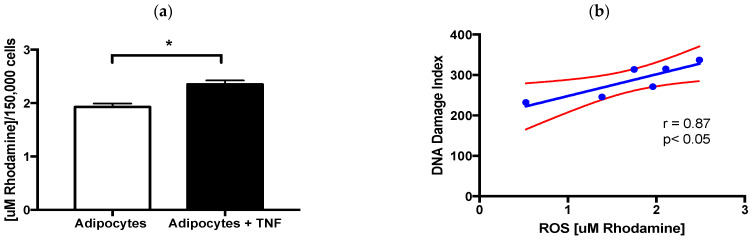
ROS and DNA damage. (**a**) Intracellular ROS levels induced by TNFα treatment (24 h, 125 ng/mL) in newly differentiated adipocytes, three independent experiments with experimental replicate were carried out. Student’s t test * *p* < 0.05. (**b**) Positive relationship between intracellular ROS levels and DNA damage index induced by TNFα. Pearson´s correlation, coefficient of correlation r = 0.87 and * *p* < 0.05.

Considering that TNFα has been related to various pathologies due to its ability to induce ROS via NADPH oxidase, particularly NOX4 [12], we went to determine the ROS levels induced by the TNFαstimulus and show its relationship with DNA damage. We found that early exposure of adipocytes to 125 ng/mL of TNFα for 24 h significantly induces ROS. Furthermore, these intracellular ROS levels correlate with the rate of DNA damage [Figure 3].

### 2.3. DNA Damage Response Activation by TNFα

Although the treatment with the TNFα was for 24 h, we found a correlation between the increase in ROS levels and DNA damage, which is one of the many possible cellular responses triggered by TNFα. For this reason, the activation of the DNA damage response in adipocytes was determined, which could indicate whether there is persistent damage that leads to the establishment of SASP.

**Figure 4:**
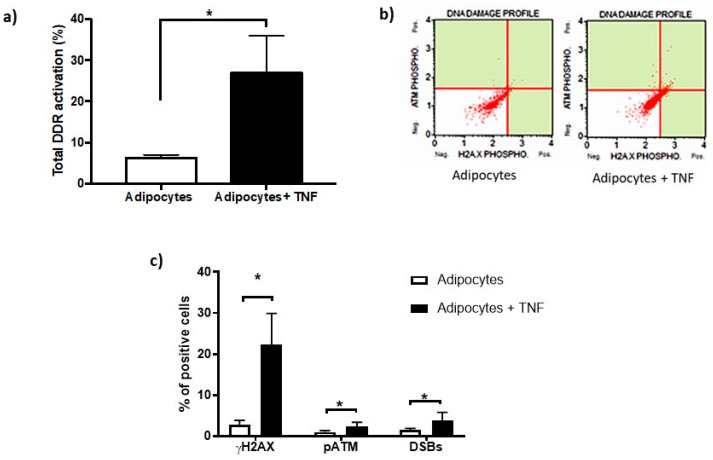
DNA Damage Response (DDR) activation basal and after-TNFα treatment. (**a**) Percentage of DNA damage response activation in adipocytes induced by TNFα (125 ng/mL, 24 h), three independent experiments with experimental replicate were carried out. Student *t* test * *p* < 0.05. (**b**) The capture of representative cytofluorometry analyzes as images that show γH2AX in X-axis, pATM in Y-axis. DSBs corresponds to the upper right signal, this means double stain. (**c**) Percentage of positive cells distribution as part of DNA damage response showing the TNFα effects. One-way ANOVA test * *p* < 0.05.

The activation of DDR by TNFα was determined by means of flow cytometry, where the percentage of cells positive for phosphorylation of DDR sensor proteins, such as histone γH2AX and ATM kinase, and DSBs that correspond to double staining (pATM and γH2AX) was quantitatively analyzed [Figure 4a,b]. However, it is important to mention that this significant increase in DDR activity represents only 25% of the cell population. Likewise, we show that DDR activation was mainly reflected by a significant increase in histone phosphorylation (γH2AX), as seen in Figure 4c.

### 2.4. SASP and Sustained DDR

**Figure 5:**
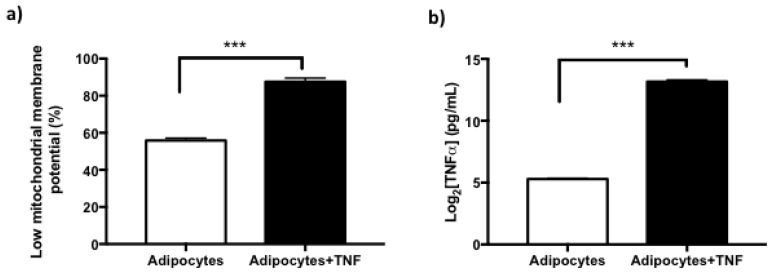
Secretory Senescence-Associated Phenotype (SASP) in adipocytes treated with TNFα. (**a**) Increase of newly differentiated adipocytes with low mitochondrial membrane potential by TNFα (125 ng/mL, 24 h) treatment, three independent experiments were carried out. Student t test *** *p* < 0.001 (**b**) Basal TNFα secretion levels in newly differentiated adipocytes and increase TNFα-secretion in adipocytes after TNFα treatment, three independent experiments were carried out. Student’s t test *** *p* < 0.001.

Considering that DDR activation is manifested mainly by the γH2AX response for at least 24 h, very likely other cellular alterations typical of SASP could be present. Based on this, we determined mitochondrial dysfunction using a semi-quantitative test for the loss of maintenance of the electrochemical potential of the inner mitochondrial membrane. It was determined that TNFα induces a 30% increase in adipocytes with low mitochondrial membrane potential, compared to untreated adipocytes [Figure 5a]. 

Additionally, even though cytokines such as TNFα have a relatively short life span (approx. 50 min) both in vivo and in vitro, they are also capable of autocrine regulation for long periods of time. With this purpose, it was evaluated whether TNFα was inducing its own regulation, quantifying the levels of cytokine secretion to the culture medium of both experimental conditions. The results indicate that there is autocrine regulation of TNFα by their determinations in culture media [Figure 5b], where it is also evident that the levels of secretion are higher in the cultures of adipocytes treated with TNFα.

**Figure 6:**
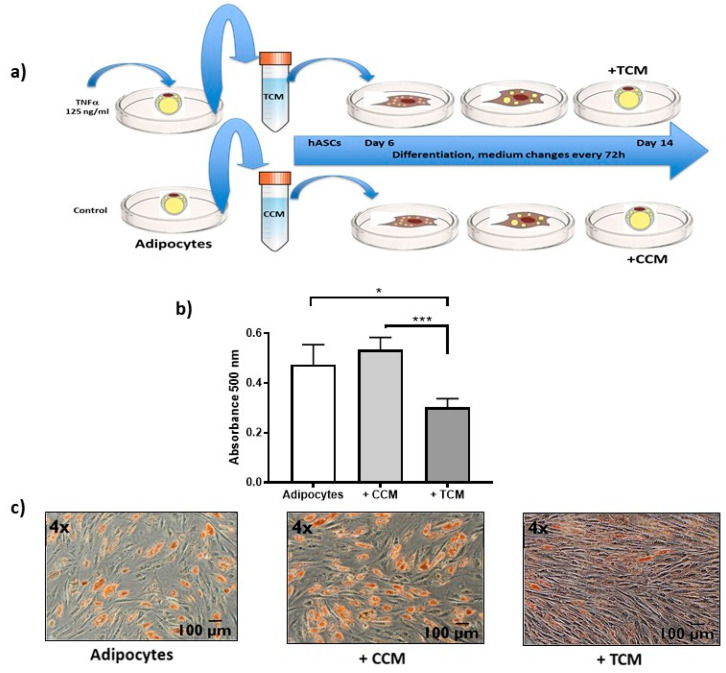
Effects of SASP on adipogenesis. (**a**) Experimental design followed to know the effect of the secretome on surrounding cells. Untreated (CCM) and TNFα-treated (TCM) adipocyte medium was harvested. The differentiation of hASCs was started six days prior in optimal conditions (as described in materials and methods) until day 6 of culture. The preadipocytes of these cultures were exposed to + CCM or + TCM and differentiation stimuli until day 14 of adipogenesis, changing the medium every 72 h. (**b**) Spectrophotometric quantification at 500 nm of absorbance of lipid droplets identified by Oil Red O staining at the end of adipogenesis, comparing control adipocytes with those treated with + CCM and + TCM. Three independent experiments were carried out. One-way ANOVA test * *p* < 0.05 and *** *p* < 0.001 (**c**) 4× magnification light microscopy images representative of Oil Red O staining. hASCs adipogenesis is not affected by secretions from untreated adipocyte (+ CCM), while the secretions of adipocytes treated with TNFα (+ TCM) limit adipogenesis showing lower staining of lipid droplets with Oil Red O.

Since DDR works through soluble signaling molecules, such as TNFα secreted to the culture medium, we determined how they affect the functioning of surrounding cells. With respect, we show evidence of the alteration in the adipogenesis from hASCs caused by the secretome of untreated adipocytes, called control conditioned medium (CCM), and that of adipocytes treated with TNFα, called treated conditioned medium (TCM) [Figure 6a]. hASCs that initiate adipogenesis were exposed to CCM or TCM since day 6 of the differentiation process, inducing a strong adipogenesis inhibition, reflected in a low stain of Oil Red O, principally induced by TCM [Figure 6b,c].

**Figure 7:**
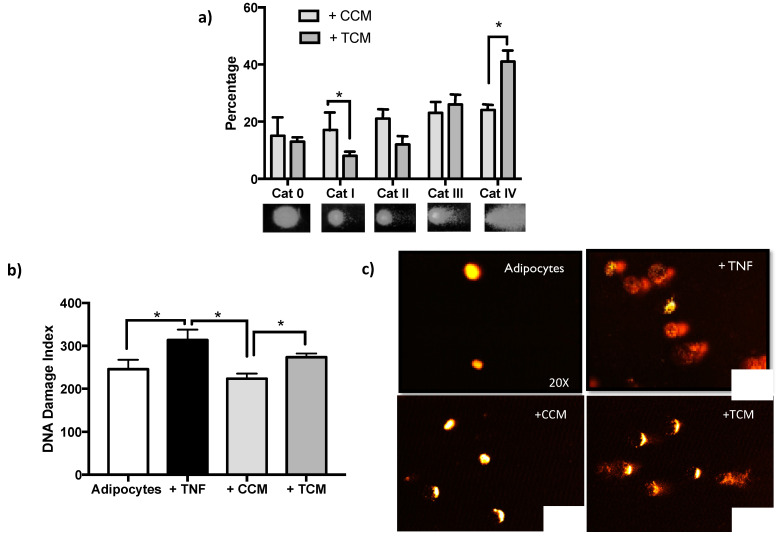
DNA damage persistent in Sustained DNA Damage Response (DDR). (**a**) Increase of the percentage distribution of DNA damage categories (Cat 0 less damage to Cat IV higher damage), after adipogenesis with Treated (TNFα) Conditioned Medium (+ TCM), three independent experiments were carried out. Two-way ANOVA test * *p* < 0.05. (**b**) The DNA damage index increases after adipogenesis in TCM relative to differentiation in CCM. The comparison shown including the basal damage of the adipocytes and adipocytes treated with TNFα from which the conditioned media come from (ANOVA multiple comparison analysis, * *p* < 0.05). (**c**) DNA damage images representatives of comet assay, acquired with Komet V5.0.

**Figure 8:**
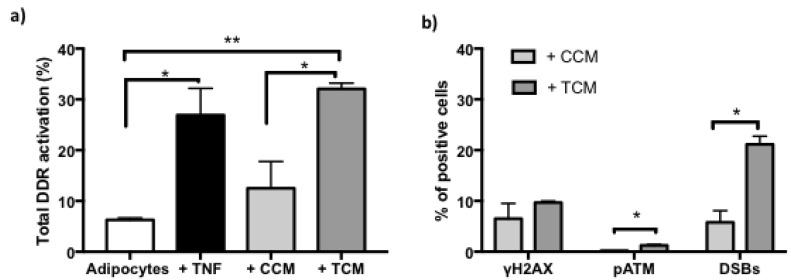
Sustained DNA Damage Response (DDR) activation vs. basal. (**a**) Increased percentage of DNA damage response activation in adipocytes treated with TNFα (125 ng/mL, 24 h), and after adipogenesis in conditioned control medium (+ CCM) or treated control medium (+ TCM). Three independent experiments with experimental replicates were carried out. Multiple comparison analysis ANOVA * *p* < 0.05, ** *p* < 0.001 (**b**) Increases in the percentage of positive cells distribution of pATM and DSBs after adipogenesis with medium conditioned treated (+ TCM). Three independent experiments were carried out. One-way ANOVA test * *p* < 0.05.

To find out if the effects caused by the secretome of adipocytes treated with TNFα affects genomic integrity after the last 8 days of the cellular adipogenesis, DNA damage [Figure 7] and sustained activation of DDR were quantified [Figure 8].

The increasing magnitude of DNA damage induced by TCM demonstrates the ability of the secretome to induce genotoxicity in a similar way to that induced by direct exposure of adipocytes to TNFα [Figure 7b], showing a persistent cellular response typical of SASP.

Persistent effects are also reflected in DDR activation, which is shown for both CCM and TCM-treated preadipocytes for 7 days [Figure 8a]. However, the induction patterns are mainly manifested by increases in double staining corresponding to DSBs, and in γH2AX kinase [Figure 8b]. These results suggest that an acute stimulus (TNFα 125 ng/mL 24 h) in newly differentiated adipocytes is sufficient to trigger autocrine and paracrine responses that generate an accumulation of DNA damage and persistent DDR that together describe the establishment of SASP.

## 3. Discussion

Adipocyte dysfunction is considered a key element in the appearance and maintenance of different metabolic pathologies, which can be initiated by intracellular pathophysiological mechanisms such as the sustained activation of stress response pathways [13,14]. Some studies have shown that the early activation of one of the stress pathways, called DDR (DNA Damage Response), precedes the loss of function of the adipocyte, although the causes and mechanisms that produce it have not been established [4,15]. Hence our interest in determining how early it is possible to identify DDR activation in adipocytes. Previously, we identified that during adipogenesis, there was an accumulation of SSBs and oxidative damage in the DNA that led to a loss of the reparative capacity of the newly differentiated adipocyte [10]. Therefore, we proposed the possibility that the chronicity of DNA damage was the necessary stress stimulus to trigger the activation of DDR in a sustained way and at the same time the door to initiate an early adipocyte pathophysiological state. DDR is a network of signaling pathways that responds to damage induced by different genotoxic agents, including products of intrinsic cellular metabolism such as ROS [2,16]. Furthermore, considering that some inflammatory stimuli, such as TNFα, were associated with the induction of DNA damage [17], we were interested in using this pro-inflammatory cytokine, that in various metabolic pathologies such as obesity and Mellitus diabetes, was found in high and constant concentrations in adipose tissue [11,18]. The present work was focused on providing an in vitro approach to the role of TNFα in newly differentiated adipocyte dysfunction, addressing their role as an inducer of oxidative stress and DNA damage, on establishing whether they lead to the sustained response of DDR.

Initially, we replicated the adipogenesis process described by our working group in 2018 [10], from hASCs, and we show evidence in Figure 1 of the arrest in the G1 phase of the cell cycle and through photomicrographs and oily red assays, the presence of lipid droplets. Although it is true that cells do not show the unilocularity of the white adipocyte, it is important to highlight that the in vivo animal differentiation process requires an entire niche or microenvironment that is difficult to replicate in an in vitro model [18]. This microenvironment is characterized by cellular heterogeneity that, through soluble and non-soluble factors, regulates the differentiation process. This morphology is very similar to that observed by other working groups, which argue that morphological change of hASCs is correlated with an increase in adiponectin secretion [19]. Even though our model has certain limitations, it represents a reliable source of information because it reduces variables typical of a 3D model and in vivo in animals. Some of these variables are the difficulty to discern between different cell populations, differential exposure to nutrients and/or treatment, and asynchronous differentiation.

Once we obtained adipocytes, we exposed them to TNFα and determined that there were no effects on cell survival after 24 h of treatment [Figure 2a], while genotoxic effects determined by the alkaline comet assay were observed [Figure 2b–d]. However, it must be considered that adipocytes per se already have an accumulation of SSBs-DNA [10], and the stimulus for the first time of TNFα at levels similar to those circulating in obese individuals [18] increases them by 25%. The SSBs-DNA determined by the alkaline comet assay is usually associated with oxidative stress, which is a mechanism of action exerted by TNFα, in such a way that we were able to establish the correlation that exists in newly differentiated adipocytes between damage to the DNA and intracellular levels of ROS [Figure 3], without this finding signifying causality. In this regard, it was reported that TNFα increases the expression of NOX4 in adipocytes due to the increase in ROS [12,20]. However, it will be important to realize some studies including antioxidants to determine the loss of this relationship. 

The persistence of intracellular ROS after 24 h of treatment highlights the signaling of intrinsic stress stimuli triggered by TNFα that, together with genotoxicity, lead to DDR. In addition to the accumulation of damage leading to DDR, this response is undertaken with the aim of promptly and efficiently repairing injuries [21,22]. Indeed, our finding was to determine that adipocytes, upon receiving TNFα 125 ng/mL for 24 h, not only presents DNA damage it also presents an activation of DDR for more than 24 h (Figure 4). An hour-to-month activation of DDR, termed sustained DDR activation, was reported to precede adipocyte dysfunction [23]. The result of the present work indicates an increase of more than three times in the total activation of DDR induced by a single stimulus of TNFα in newly differentiated adipocytes. This is derived from considering the phosphorylation of ATM and H2AX kinases (γH2AX), which are widely accepted as markers of DDR activation [24,25]. It should be noted that as part of the activation of DDR, 85% of the positive response corresponds to γH2AX. This result may suggest several scenarios, (i) early DDR signaling since γH2AX favors the recruitment of DDR effector proteins; (ii) and the sustained response of DDR, since a transient DDR occurs with early dephosphorylation of γH2AX (H2AX), from minutes to a few hours [4]. This can be corroborated by monitoring DDR activation for several days after treatment with TNFα to identify if the values determined in adipocytes are recovered.

Although the activation of DDR supposes the preservation of genomic integrity, sustained activation favors processes such as senescence and cellular dysfunction, in this case of the adipocyte [26]. In the results shown, we did not specifically evaluate senescence. However, quantifying the activity of the lysosomal enzyme β-galactosidase can corroborate this. What we do find in our results are indicators of adipocyte dysfunction through SASP markers, such as increase in ROS levels, DNA damage, low mitochondrial membrane potential, and increased secretion of pro-inflammatory cytokines (TNFα) in adipocytes treated with the stimulus obesogenic [Figure 5]. We are aware that the adipocyte secretome requires validation by the identification of more several cytokines as IL1-β, IL-6, metaloproteinases, EGF (epidermal growth factor) principall. However, we could not make these determinations in the present work, since we require the medium to treat other cultures in the process of differentiation. Therefore, as a perspective, the determination of a variety of cytokines should be carried out as part of the characterization of the secretome. It is worth mentioning that the present work is a pioneer in showing evidence of the sustained activation of DDR mediated by TNFα in newly differentiated human adipocytes. For this reason, it was important to try to identify how this sustained response of DDR affects neighboring cells, considering that the secretion of TNFα into the environment represents an autocrine regulation [27].

In an in vivo animal model, the acquisition of SASP allows senescent cells to promote processes such as tissue restoration, cell differentiation, proliferation, and even the activation of DDR in adjacent cells [2,3]. However, pro-inflammatory secretions such as TNFα have also been reported to inhibit the adipogenic process in hASCs cells [28]. In the present work, we present evidence that cellular processes as SASP and adipogenesis alterations are triggered by exposure to TNFα in adipocytes [Figure 6, Figure 7 and Figure 8]. On the one hand, we show evidence that the secretome of adipocytes treated with TNFα is capable of partially inhibiting the differentiation of preadipocytes exposed to the treated conditioned medium (TCM), at the same time that we observe an increase in the cell density of the cell cultures. We propose that this observation could mean the presence of proliferative stimuli triggered by SASP [Figure 6c]. For this purpose, analysis of proliferation and cell cycle could be performed. In this regard, it has been reported that the inhibitory effect of differentiation begins with the internalization of the signal through TNFR1 [29], which inhibits PPRγ through NFκB. However, the information about this adipogenic inhibition is limited in compromised cells, such as the preadipocytes that we use in the present work.

As mentioned above, we also identified the induction of DNA damage by TCM in treated preadipocytes [Figure 7b]. The magnitude of the damage was less than that induced by the direct exposure of adipocytes to TNFα, which we suggest is due to the fact that by having a certain proportion of cells with high proliferation capacity, they have more efficient repair mechanisms that are capable of counteracting the damage. To confirm this, some proliferation markers such as Ki67 or PCNA could be measured. Alternatively, this result suggests that the levels of pro-inflammatory cytokines are different; therefore, it is important to consider the possibility of characterizing the content of TCM.

There is no doubt that the sustained activation of DDR induced by TNFα exerts effects on neighboring cells through the establishment of SASP. What adds relevance to this finding is that the activation of DDR in neighboring cells is even greater than that observed by the effect of TNFα in adipocytes and is mainly caused by an increase in cells positive for DSBs and γH2AX. This suggests that the TCM directly induces DSBs or processes such as translesion synthesis (TLS) are induced through cells with high proliferation. However, it is also likely that the adipocyte damage recording mechanisms differ from that of preadipocytes. In this regard, it has been reported that when proliferating fibroblasts are exposed to ionization, radiation is identified in the medium pATM and 53BP1 and that this pATM functions as a differentiation inhibitor [30,31]. If so, it is feasible to determine DSBs by alternative methodologies and the presence of 53BP1 as well. The scenarios discussed above have canonical perspectives, however, recently, Lee and Paull [32] reviewed the relevance of ATM in various human diseases. These authors proposed different ways of regulation of ATM through ROS, mitochondrial dysfunction, alterations in transcription, defects in cellular proteostasis and metabolism, among others, which start with DNA-SSBs as a lesion induced by several oxidative stress cellular environments. SSBs have not been a focus in ATM research as nicked DNA does not activate ATM in vitro, although recent findings showed that ATM could be activated in cells with deficiencies in SSB repair or in cells exposed to oxidative stress and alkylating agents, which can lead to SSBs. This information confers relevance to the present study because we identified in an in vitro model ATM activation by SSBs induced by TNFα, indicating their relationship with mitochondrial dysfunction of the adipocytes; in addition to our previous work that show the loss in DNA repair of SSBs in adipocytes.

As a whole, the present work shows how a newly differentiated adipocyte acquires cellular dysfunction, related to the sustained activation of DDR and due to the effects caused to neighboring cells, indicates the possible set of events that initiate the establishment of a pathophysiological state beyond and those triggered by aging.

## 4. Materials and Methods

### 4.1. Cell Culture

For the present work, mesenchymal stem cells derived from human adipose tissue, hASCs (ATCC^®^ Cat. No. PCS500011™) were used. The cells in passage 3−4 were seeded in polystyrene culture boxes, where the culture medium “MesenPRO RS™ Medium GIBCO” (Cat. No. 12746-012) prepared according to the supplier’s specifications was used. They were kept in incubation at 37 °C with 95% humidity and 5% CO_2_. Every 48 h, there was a change of culture medium until they reached a confluence of 80−85%. For harvesting, the culture was treated with 0.05% Trypsin-EDTA (Gibco Cat. No. 25300-054) for 3 min at 37 °C and 5% CO_2_ to obtain a total cell suspension. Cells were transferred to PBS and centrifuged at 300× *g* for 5 min. 

### 4.2. Cell Differentiation

Once the cultures reached 85% confluence, the differentiation stimulus towards adipocytes was given. MesenPRO medium was discarded and washed with sterile 1X PBS, and differentiation medium “StemPro^®^ Adipogenesis Differentiation Kit, Gibco” (Cat. No. A10070-01) was supplied. The medium was prepared following the supplier’s instructions. They were kept in incubation at 37 °C with 95% humidity and 5% CO_2_. Every 72 h, there was a change of fresh medium (StemPro^®^) until they reached the desired day of differentiation, day 14. Subsequently, the cells were harvested following the protocol described for hASCs, which varies only in the concentration of trypsin used 0.25% Trypsin-EDTA (Gibco, Cat. No. 25200-056).

### 4.3. Oil Red O Stain

To confirm adipocyte differentiation, the hASCs were seeded in 12-well plates. Differentiation was initiated when confluence was 80%. The corresponding stains were carried out at days 0, 7, and 14 of differentiation. Cells were washed twice with PBS and then fixed with 10% paraformaldehyde for 30 min at room temperature (RT). After that, 2 washes with PBS were performed, and Oil Red O (Trevigen Cat. No. 5010-024-05) prepared according to the supplier was added, followed by incubation for 30 min with gentle shaking and light protection. Finally, PBS washes were performed twice, and cells were observed at an inverted microscope (Olympus IX50-S8F2) to acquire images with magnifications of 4x and 40x. For stain quantitation, the PBS was removed, and dye was extracted with isopropanol (750 μL) and incubated for 10 min with shaking and light protection. The supernatant was collected, and absorbance of the sample was measured on a spectrophotometer (Ultraspec 3000), Pharmacia Biotech) at 500 nm using isopropanol as a blank to obtain the relative lipid accumulation.

### 4.4. Cell Cycle Analysis

The cell cycle was evaluated using a DNA intercalating agent (propidium iodide; IP) and RNaseA to decrease non-specificity (Muse Cell Cycle Kit, Cat. No. MCH100106). A total of 10^5^ cells were collected in a tube to be centrifuged at 300× *g* for 5 min and washed with PBS. Ethanol 70% was added to suspend cells in a concentration of 10^5^ cell/mL. It was incubated for at least 3 h at −20 °C. A total of 200 μL of fixed solution in a new tube was added and newly centrifuged for 5 min at 300× *g* and washed once with PBS. Then 200 μL of Muse Cell Cycle reagent was added and incubated for 30 min at room temperature in the dark. Realize cytometer acquisition in Muse^®^ Cell Analyzer Cat No. 6500-3115.

### 4.5. Cell Viability

A lysosomal activity test was used to determine cell viability and was performed using the cFDA-EtBr (5, 6 carboxyfluorescein diacetate-ethidium bromide) stain. The cell suspension was mixed 1:1 with stain solution (20 μL of EtBr (0.02 mg/mL) and 3 μL of cFDA (0.015 mg/mL) prepared in fresh), and the analysis was performed by fluorescence microscopy (Olympus BMX-60 with a UM61002 filter) at 20× magnification. cFDA is taken up by cells that, through esterase activity, transform no fluorescent cFDA into a green, fluorescent metabolite. The nuclei of the dead cells were stained with ethidium bromide and visualized as red fluorescence.

### 4.6. TNFα Treatment

Once day 14 of differentiation was reached, adipocytes were washed with 1x PBS, to remove cell remnants. The cells were exposed 24 h to TNFα at a concentration of 125 ng/mL. The concentration was taken from that reported by Lo et al. [11] and Turner et al. [18] as an obesogenic stimulus, as it represents the circulating TNFα levels in obese patients with adipocyte dysfunction. The control condition only consisted of a change of medium. After the treatment time, the cells were subjected to the different established tests.

### 4.7. Comet Assay

DNA damage was determined by comet assay to evaluate the presence of DNA breaks produced, which includes single and double-strand breaks, as well as alkali labile sites in adipocytes from hASCs. For each experimental condition, at least 10,000 cells were mixed with 75 μL of 0.5% low melting point (LMP) agarose. The cells were loaded onto microscope slides pre-layered with 200 μL of 0.5% normal melting point agarose and covered with a third layer of LMP agarose 0.5%. Briefly, after lysis of the cells at 4 °C for at least 1 h in a buffer consisting of 2.5 M NaCl, 100 mM EDTA, and 10mM Tris, pH10, supplemented with 10% DMSO and 1% Triton X-100, the slides were placed in a horizontal electrophoresis chamber with running buffer solution (300 mM NaOH, 1 mM Na2EDTA, pH > 13). The slides remained in the electrophoresis buffer for 20 min to allow the DNA to unwind and reveal alkali-labile sites (AP sites). Electrophoresis was performed for 20 min at 300 mA and 25 V, ~0.8 V/cm. All steps were performed in the dark to avoid direct light. After electrophoresis, the slides were gently removed and rinsed with neutralization buffer (0.4M Tris, pH7.5) at room temperature for 10 min, dehydrated with absolute ethanol for 15 min, and air-dried. Ethidium bromide (20 μL of 20 mg/mL solution) was added to each slide and a coverslip was placed on the gel. Individual cells were visualized at 20× magnification with an Olympus BX-60 microscope with fluorescence attachments (515–560 nm excitation filter, 590 nm barrier filter), and the DNA damage was determined using Komet V5.0 software (Kinetic Imaging Ltd., Budapest, Hungary). To evaluate DNA damage, 50 nucleoids per slide, 2 slides per experiment, and 3 experiments (300 nucleoids total per condition) were scored for each experimental condition. Nucleoids images acquired were divided into 5 categories according to the damage categories, Cat 0- non-damage; Cat I-low damage; Cat II-medium damage; Cat III-high damage, and Cat IV-very high damage. The total number of nucleoids in each category was counted and multiplied by an assigned value of 0–4 according to the damage class. The sum of all the categories was calculated and considered the damage index. The overall score was expected to vary between 0 and 400 arbitrary units. Damage index = (% of Cat 0) × 0 + (% of Cat I) × 1 + (% of Cat II) × 2 + (% of Cat III) × 3 + (% Cat IV) × 4.

### 4.8. Reactive Oxygen Species (ROS)

The principle of this technique is based on the reading of the fluorescence emitted by the oxidation of dihydrorhodamine-123 (Calbiochen, Cat. 309825) to rhodhamine, depending on the intracellular concentration of ROS. From the cells previously harvested, a volume of cell suspension containing 150,000 cells was taken, which was centrifuged at 1200 rpm for 5 min, and the supernatant was discarded. 180 µL of solution A (140 mM NaCl, 5 mM KCl, 0.8 mM MgSO4, 1.8 mM CaCl2, 5 mM Glucose and 15 mM HEPES), and 20 µL of stock solution of dihydrorhodamine-123 (1 µM) were added to the cell button, the mixture was carefully suspended and transferred to a 96-well plate for fluorescence, where it was allowed to incubate at 37 °C for 2 min. The plate was read using a Thermo Scientific Multiskan GO fluorometer (Cat. No. 51119200) with a 505 nm excitation filter.

### 4.9. Activation of DNA Damage Response, DDR

To evaluate DDR activation, the Muse Multi-Color DNA Damage Kit (Cat. MCH200107) was used following the supplier’s instructions. The assay consists of two directly conjugated antibodies: a phospho-specific ATM (Ser1981) -PE and a phospho-specific histone H2A.X PECy5-conjugated antibody to measure activation of the DNA damage response. This two-color kit is designed to simultaneously detect the phosphorylation status of ATM and Histone H2AX by flow cytometry analysis. At the same time, quantify DSBs to understand the mechanisms involved in DNA repair and the response to DNA damage. The harvested cells were centrifuged at 1200 rpm for 5 min, and the supernatant was discarded. A 100 µL volume of a 1: 1 mixture of assay buffer (1×) and the fixative solution was added to the cell button to be carefully suspended and allowed to stand for 10 min on ice. Subsequently, the cells were centrifuged at 1200 rpm for 5 min, and the supernatant was discarded, and a volume of 100 µL of permeabilization buffer was added for 10 min on ice. At the end of this period, the cells were centrifuged at 1200 rpm, for 5 min, the supernatant was discarded. To the cell button, 90 µL of assay buffer (1X) and 10 µL of a 1: 1 solution of antibodies (ATM and H2AX) were added, and it was left to stand for 30 min at room temperature, in the dark. 100 µL of assay buffer was added to the cells, and they were centrifuged at 1200 rpm, for 5 min, and the supernatant was discarded. Finally, the cell button was suspended in 200 µL of assay buffer to later be quantified using the Muse^®^ Cell Analyzer Cat No. 6500-3115 cytometer. Data generated by the Muse^®^ Cell Analyzer along with the corresponding Muse software module provides statistical values measuring: Percentage of negative cells, percentage of ATM activated cells, percentage of H2AX activated cells, and percentage of DNA-DSBs (dual activation of both ATM and H2AX).

### 4.10. Detection of Mitochondrial Membrane Potential

In any cell type, the mitochondria play a vital role in the proper functioning of cellular metabolism. Faced with constant stress, such as high levels of ROS, the mitochondrial membrane becomes permeable, and the electrochemical gradient of the mitochondrial membrane collapses. In this way, the cell is unable to carry out its metabolism at optimal levels and, in the worst case, is induced to apoptosis through the release of cytochrome c and the activation of caspases. To determine if there is a mitochondrial dysfunction that allows us to assume a malfunction of the adipocyte, the disruption of the mitochondrial membrane potential was analyzed. This test was performed using the Muse MitoPotential Kit (Cat. MCH100110). Initially, the Muse MitoPotential working solution was prepared by diluting MitoPotential Dye in 1X assay buffer (1:1000). Subsequently, the cells were harvested in serum or medium with albumin in a concentration of 100,000 to 500,000 cells/mL. Cells were centrifuged and suspended in 1X assay buffer. Then, 100 µL of cell suspension was added to a tube and 95 µL of MitoPotential working solution, mixed with a pipet for 5 s and incubated for 20 min, at 37 °C in a CO_2_ incubator. Then, 5 µL of 7-AAD reagent was added, mixed with a pipet for 5 s, and incubated for 5 min at room temperature. Finally, the fluorescence was read using the Muse^®^ Cell Analyzer Cat No. 6500-3115 cytometer.

### 4.11. TNFα Secretion

The half-life of TNFα, both in vivo and in vitro, is relatively short, approximately 50 min. However, it is known that the presence of TNFα can remain for long periods through self-regulatory processes. To quantify the concentration of TNFα secreted into the culture medium, an ELISA was performed. This assay was performed according to the supplier’s instructions (Quantikine^®^QuicKitTM ELISA, RD Systems Cat. KQ210). This medium was collected from newly differentiated adipocytes pretreated with 125 ng/mL of TNFα for 24 h and untreated adipocytes. The procedure consisted of adding 50 µL of medium per well and over it, adding 50 µL of the antibody cocktail and protecting from light. Subsequently, it was incubated for 1 h at room temperature on a microplate shaker, each well was aspirated, and 3 washes were carried out with 1× PBS. Subsequently, 100 µL of substrate solution was added to each well and incubated for 20 min at room temperature and protected from light. Finally, 50 µL of blocking solution was added to each well and incubated for 30 min to read the plate by means of Thermo Scientific Multiskan GO (Cat. 51119200). In the plate reading, the optical density of each well was determined at 450 nm. In addition, a wavelength correction was made at 540 nm, which was subtracted from the readings at 450 nm, as indicated by the supplier. The determination of a standard curve was carried out and the data obtained were interpolated to be expressed in pg/mL.

### 4.12. Statistic Analysis

All the tests, previously described were carried out in 3 independent experiments with experimental replication. Once this minimum n number was obtained, a statistical analysis was performed using parametric tests. This was because the sample data behaved in a Gaussian manner. These analyzes were ANOVA (One-Way ANOVA on Ranks), Turkey’s test, and Student’s t test. These tests were carried out with the help of the GraphPad Prism 6 software. To rule out random differences, a value of * *p* < 0.05, ** *p* < 0.01, *** *p* < 0.001 was taken as the significance parameter.

## 5. Conclusions

A single stimulus of 125 ng/mL of TNFα, per 24 h does not affect the cell viability of newly differentiated adipocytes from hASCs cells. However, it increased single-strand breaks in DNA, as well as intracellular ROS levels. A positive, simple relationship between DNA damage and ROS was evidenced without being able to determine whether the relationship is causal. The present work is a pioneering study in showing evidence of induction of sustained activation of DDR in newly differentiated adipocytes due to TNFα exposure. In addition to identifying the establishment of SASP by showing the effects of the secretome on preadipocytes, evidencing its ability to inhibit adipogenic differentiation partially. It should be noted that the magnitude of cellular damage triggered by just an obesogenic stimulus in newly differentiated adipocytes shows the vulnerability to the establishment of pathophysiological states in adipocytes, regardless of aging.

## Data Availability

Not applicable.
